# Recovering from burnout: Doing a home visit and finding an old friend and an entire community

**DOI:** 10.4102/safp.v65i1.5750

**Published:** 2023-05-15

**Authors:** Sheron T. Forgus

**Affiliations:** 1 Private Practice, Cape Town, South Africa

It was that dreaded year 2020 that broke humankind. Like many of us, its energy forced its way through the cracks in my resolve and split me wide open. After months of coronavirus disease (COVID-19) pandemic warfare as a family physician in a gang-ridden, poverty-stricken community, like many if not all of us, I was struggling. I had just buried my father and my mother was fighting for her life against COVID-19 in hospital. My body (and husband) said: Enough.

In COVID-19’s defence, burnout and the loss of this doctor’s sense of purpose didn’t happen in a year. It was a poisonous potion I drank slowly, bit by bit over the preceding 5 years as I took on more than I could carry and tried to fix everything that was broken.

After I resigned, I spent months lying on the couch, too tired to do anything but send my kids off to school and welcome them home again. I swallowed the pills, I did the sessions and I read the books, but I did not have a plan. All I knew was I did not want to see a patient ever again. Something had died in me and compassion felt like a banana that had rotted at the bottom of my medical bag months ago.

I was worried that we would reach the end of the financial buffer and I would be forced back into the system and end up right where I was before. Part of me also wanted to know whether medicine and I were really over. This was how it came about that I started working half days at a private group practice after a year and a half.

And, just like the coach said, slowly but surely, patient by patient, I started to thaw. Without the stress of managing a facility, governing a system, supporting staff and running on the treadmill of admin and meetings, I found myself connecting with patients again and the ball of tension unravelled. I felt something stirring inside and I could not quite place it.

Until Mr. GG walked into my office one day. He was old-school cool. The kind that dresses up for his doctor’s visit wearing a pullover, a shirt and freshly shined shoes. He was a polite and eloquent, retired gentleman who had even booked his own podiatry visit to have his diabetic feet taken care of. If love was a patient, he was it. After Mr. GG left, I felt something stirring in me again. Golden threads of something tingling inside of me …

Two weeks later that same man walked into our practice unable to speak as new-onset atrial fibrillation and a fresh cerebrovascular accident presented itself. I stabilised Mr. GG and sent him to the hospital. A few days later and many thereafter, I tried to reach him on his cell phone, but it gave no answer. I feared the worst and I did not know why I was not ok with not knowing.

It was 2 weeks before I forced myself to listen to the tiny voice inside, which told me to check the address – it was less than a kilometre away.

I do not know who was happier to see whom that day. That afternoon, I did not do a home visit in the conventional sense of the word. I had neither medical bag nor records with me. I offered no advice and they sought none. I sat in a chair next to his bed and I listened to them tell their story about what had transpired over the last 2 weeks, and I simply said how happy I was to see they were both all right. As Mr. GG walked me to the gate and thanked me, I felt like it was me who should be thanking them. As the sun set that day I smiled as I realised I went looking for my patient, but I found someone far dearer to me instead. Hello compassion, my old friend …

Rediscovering my purpose also meant I wanted to help other family physicians avoid a similar experience to mine. This led me to become a mentor for the Next 5 – an initiative by the South African Academy of Family Physicians (SAAFP) that supports family physicians in their first 5 years after graduating.^1^ Through the mentorship programme, newly appointed family physicians will benefit from an active process of support and role clarification within their healthcare teams to establish themselves in the health system and mature in all their different roles.

If you are experiencing burnout or in need of self-care, joining a mentorship programme will enable you to connect to a community of care and practice, whether you are based in the public or the private sector. Existing family physicians can join the initiative as mentors just as I have done. Through this, we can create an ongoing ‘sense of belonging’ as I experienced within the SAAFP community.
